# Benefits of Early Enteral Feeding with a Locally Prepared Protein-Energy Ration after Surgery for Acute Generalised Peritonitis: A Randomised Trial in Two Hospitals in Bukavu, Eastern Democratic Republic of Congo

**DOI:** 10.1155/2023/1764242

**Published:** 2023-11-17

**Authors:** Jean Paul Buhendwa Cikwanine, Jonathan Tunangoya Yoyu, Désiré Munyali Alumeti, Bernard Mugisho, John Mutendela Kivukuto, Rivain Fefe Iteke, Ona Longombe Ahuka, Willy Kalau Arung

**Affiliations:** ^1^Faculty of Medicine, Evangelical University in Africa, Bukavu, Democratic Republic of the Congo; ^2^Panzi Hospital, Bukavu, Democratic Republic of the Congo; ^3^International Centre for Advanced Research and Training, Bukavu, Democratic Republic of the Congo; ^4^Progressive Medical Systems/Department of Works and Medical Research, Goma, Democratic Republic of the Congo; ^5^Rau Ciriri Hospital, Bukavu, Democratic Republic of the Congo; ^6^MDA, Savigny-Sur-Orge, France; ^7^University of Lubumbashi, Lubumbashi, Democratic Republic of the Congo; ^8^Department of Surgery, University of Kisangani, Democratic Republic of the Congo

## Abstract

**Background:**

Acute generalised peritonitis (AGP) is a common and serious digestive surgery pathology. Undernutrition exacerbates patient condition and compromises their postoperative prognosis. Early enteral nutrition is recommended to reduce postoperative complications, but its availability and cost are problematic in low-income countries. The objective of this study was to evaluate the impact of providing early enteral feeding (EEF) to postoperative patients with intestinal perforation AGP using a locally prepared protein-energy food ration in two hospitals in Bukavu, a city of South Kivu, in the eastern part of the Democratic Republic of Congo.

**Methods:**

A prospective, randomised controlled trial with two groups of patients was conducted to investigate the effects of EEF with a local mixture versus enteral feeding after peristalsis had returned (control group) in patients who underwent laparotomy for AGP caused by ileal perforation. The local mixture consisted of soybean, maize, white rice, and pineapple. The trial included 66 patients with ileal perforation peritonitis.

**Results:**

The results comparing early enteral fed and nonfed patients showed significant differences in peristalsis recovery time (2.1 (0.6) days vs. 3.8 (1.2) days, *p* < 0.0001) and length of hospital stay (25.5 (14.9) days vs. 39.4 (25.3) days, *p* = 0.0046). Bivariate analyses indicated a significant early enteral feeding (EEF) reduced of 9.1% (vs. 36.4%, *p* = 0.0082) in parietal infections and 3.4% (28.1%, *p* = 0.009) in fistulas (*p* = 0.009) when EEF was included. In addition, EEF significantly reduced reintervention rates by 9.1% (*p* = 0.0003) and eliminated evisceration rates. EEF was also shown to reduce the incidence of malnutrition by 63.6% (*p* < 0.0001). Multivariate analysis showed that enteral nutrition significantly reduced the time to recovery of peristalsis (*p* = 0.0278) with an ORa of 0.3 and a 95% CI of 0.1-0.9. Moreover, EEF reduced malnutrition (*p* = 0.0039) with an ORa of 0.1 and a 95% CI of 0-0.4.

**Conclusion:**

EEF with locally sourced protein-energy rations can enhance a patient's nutritional status and facilitate postoperative recovery. This procedure is advantageous and involved early enteral nutrition using locally manufactured rations, especially for those operated on for acute generalised peritonitis in the Democratic Republic of Congo.

## 1. Introduction

Acute generalised peritonitis (AGP) resulting from intestinal perforation is a frequently occurring pathology in surgical settings, with a frequency ranging from 19% to 25%. It is the third most common emergency in digestive surgery. The primary causes of peritonitis in tropical regions are typhoid fever and acute appendicitis [1, 2]. Patients with intestinal perforation peritonitis hold a significant status as they are already influenced by the pathology that caused the perforation. Performing enteral nutrition in these patients can be challenging due to the potential risk of gastrointestinal dysmotility. This may result in distension, high gastric volumes, vomiting, and diarrhoea [3, 4]. A study demonstrated that to achieve optimal healing and functional recovery, metabolic response is necessary, which can be difficult to achieve without nutritional therapy. This is particularly true when the patients are undernourished, and the stress/inflammatory response is prolonged. Therefore, nutritional therapy is essential [5]. The detrimental impact of insufficient calorie and protein intake on the recovery of surgical patients has been demonstrated [6, 7]. Furthermore, the exacerbation of this impact is compounded by the infectious conditions in which patients are admitted, as well as hypercatabolism resulting from inadequate nutritional status and prolonged pre- and postoperative stress [6, 7].

Numerous randomised controlled trials and meta-analyses, which including studies comparing oral or enteral nutrition to nihil per os, suggest that there is no advantage to maintaining patient fasting following elective gastrointestinal resection [3, 6, 7] or major gynaecological surgery [8]. Over recent years, enteral nutrition has emerged and is grounded on a physiological rationale. Achieving the preservation of trophicity in intestinal villi, maintenance of digestive mucosa integrity and function, prevention of bacterial translocation, optimal substrate utilization, and promotion of glucose tolerance are necessary goals. Postoperative complications, including delayed healing, postoperative rehabilitation delay, and longer hospital stays, are independently increased by preoperative undernutrition [9].

The province of South Kivu in the Democratic Republic of Congo is afflicted with economic challenges, persistent insecurity, and inadequate health coverage [10, 11]. Consequently, most hospitals face a significant obstacle in providing artificial nutrition, as these products are often imported and expensive in comparison with the residents' income. For instance, enteral nutrition has an average cost of USD 170 per patient daily, while parenteral nutrition costs around USD 308 per patient per day. Meanwhile, the majority of patients earn less than USD 100 per month [9, 11, 12]. Furthermore, some surgeons are hesitant to provide early postoperative feeding to these patients due to the potential intolerance it may cause in the inflamed digestive tract. The early provision of enteral nutrition using protein-calorie products within 24 hours of surgery, as suggested by Berré and Chardon [4], may represent a promising prognostic factor in the management of these patients. The aim of this study was to assess the impact of providing early enteral nutrition using a food mixture manufactured locally on patients who underwent surgery for intestinal perforation peritonitis.

## 2. Methods

### 2.1. Study Design

This study was conducted in two hospitals located in Bukavu city, namely, Rau de Ciriri Hospital and Panzi Hospital. These hospitals were selected based on the acceptance of their surgical team to participate in the study and the presence of a senior surgeon and a trained anaesthetist within the hospitals.

A randomised controlled trial consists of two arms: an intervention arm in which patients were given early enteral feeding (intervention group) and a control arm receiving enteral feeding after the return of peristalsis (control group: traditional). The trial was conducted with patients who underwent laparotomy for acute generalised peritonitis caused by small bowel perforation.

### 2.2. Study Population and Sampling

Our study cohort consisted of 256 patients who underwent surgery for acute generalised peritonitis due to nontraumatic small bowel perforation. Out of the total population, 186 patients did not fulfil the inclusion criteria of the study while 2 opted out of participation. Considering the formula provided by Cochran [13], Harouna et al. [1] found a prevalence of 6.65% for acute generalised peritonitis. With a margin of error of 6%, a sample size of 66 patients was calculated and divided into two groups (the study group and the control group). Patients from Panzi Hospital comprised 43.9% of the selected sample, whereas those from Ciriri Hospital represented 56.1%.

The study included patients with a quick Sepsis-related Organ Failure Assessment (qSOFA) score of less than 3 and three or fewer perforations who underwent small bowel suturing. The qSOFA score is a tool used to assess the chances of mortality and severe complications in infected individuals. Those with a high qSOFA score, requiring intensive care and more complex surgical intervention, were excluded from this research. Patients with multiple perforations that required a digestive stoma, impeded the administration of early enteral nutrition, or could impair nutrient absorption from the intestine were not considered. Furthermore, patients who did not provide their informed and voluntary consent and had comorbidities that may impact the assessment of the advantages of early enteral nutrition, such as allergies to the considered products, Crohn's disease, pancreatic disorder, HIV/AIDS, diabetes, tuberculosis, liver dysfunction, cardiovascular disease, and renal failure, were not included.

### 2.3. Randomisation

Randomisation occurred after participant recruitment, with a 1 : 1 ratio between the intervention and control groups. Patients' consents were obtained prior to the randomised allocation. Subsequently, the decision to initiate early enteral feeding for the first patient was determined randomly using the Bernoulli test, with the same procedure applied to the second patient to maintain the 1 : 1 ratio.

### 2.4. Procedures and Interventions

The administration of a postsurgery oral diet involved a protein-calorie mixture being administered via a nasogastric tube for a 24-hour period. This protein-calorie mixture consisted of 75 g of soya meal, 100 g of maize meal, 100 g of white rice meal, 15 g of sugar, and 100 g of pineapple to prepare a porridge with an energy value of 1,148 kcal in 1,200 ml so that it could be administered continuously through the nasogastric tube to prevent abdominal distension. The selection of these components was based on their nutritional content, local dietary practices, and availability. Patients were administrated 20 to 25 kilocalories per kilogram per day of the final porridge composition during the acute phase, followed by 25 to 30 kilocalories per kilogram per day after stabilisation as it is recommended [14]. To prevent preservation issues, the nutritionist prepared a 24-hour batch every morning. The evaluation of nutritional status took place on day 0, which involved assessing weight, height, brachial circumference, body mass index, and blood albumin. Regular evaluation was carried out on day 5 and day 10.

During preoperative preparation, all patients underwent identical preparation regardless of their group. A multiparameter monitor was used by the nurse to record and monitor vital signs. Both groups underwent systematic placement of a bladder and nasogastric probe. To monitor hypovolemia, biological monitoring was performed at the haematocrit level as gasometry was unavailable to measure lactate levels. Blood cultures were collected routinely regardless of temperature to avoid any delay in the administration of antibiotics. Other biological tests performed routinely upon admission included a complete blood ionogram, urea and creatinine levels, blood cell count, blood glucose, albumin levels, Widal-Felix's test, and HIV serology.

All patients underwent surgery under general anaesthesia, while adhering to the recommendations on anaesthesia for a full stomach. For pain management, a bolus of ketamine 0.25 mg per kg and fentanyl two (2) *μ*cg/kg were administered. Both groups were also given a dexamethasone 8 mg bolus during induction to prevent postoperative nausea and vomiting. Ciprofloxacin 400 mg + metronidazole 500 mg was administered intravenously as probabilistic antibiotic therapy. The treatment took a maximum of 10 days following the outcomes of the antibiogram. The use of ciprofloxacin was abstained in the case of children.

Before the patient regained consciousness, all requirements for extubation were thoroughly examined. Paracetamol was administered for analgesia at a dosage of 15 mg per kilogram for children and 1 g through intravenous route for patients over 45 kg, alongside nefopam at a dosage of 20 mg through gradual infusion over a period of 15 minutes, resulting in a total dose of 120 mg per 24 hours. Postoperatively, the administration of analgesia was based on the Simple Verbal Scale (SVS), with morphine given subcutaneously in doses of 5 to 10 mg when SVS > 2.

After 24 hours, the studied group received an enteral nutrition ration tailored to daily patient needs. In the traditional group, transit had to return and a lack of hydroaerobic level on the unprepared abdominal X-ray before allowing liquid feeding. The patient could consume solid food 24 hours after initiating liquid intake. To maintain fluid, electrolyte, and energy levels, 5% saline and 1.5 to 2.5 liters per day, along with 50 to 100 mmol NaCl per day and 40 to 80 mmol KCl per day, were administered parenterally until feeding was permitted.

Measures were taken to prevent paralytic ileus and facilitate transit from an anesthetic point of view. These measures included limiting the use of morphine and its derivatives, promoting rapid mobilisation of patients postoperatively, and systematically administering a prokinetic from the 24^th^ hour in the study group. Furthermore, a blood ionogram was carried out to avoid any ileus caused by hypokalaemia. Feeding-associated adverse events entail vomiting, diarrhoea, constipation, gastroesophageal reflux, abdominal overdistention, inhalation pneumonitis, and lasting pain. In case of deterioration of adverse events, despite intervention, feedings were terminated.

### 2.5. Ethics and Consent

Approval was obtained from the ethics committee at the University of Lubumbashi (reference UNILU/CEM/166/2018), and informed consent was obtained from all participants involved in the study. Only the research team had access to the collected data.

### 2.6. Data Analysis

Descriptive statistics were performed on the sociodemographic variables, depending on whether they were qualitative or quantitative. The means of the quantitative variables between the two groups were compared using the Student *T*-test. The association between the intervention groups (study group and control group) and qualitative variables in this study was tested using Pearson's test of independence or Fischer's exact test, based on theoretical numbers greater than or equal to 5 or less than 5, respectively. The significance level was set at *p* < 0.05. A logistic regression model was performed to adjust for the effects linked to the use of early enteral feeding with local products, including variables significantly associated with the groups. The coding and analysis were carried out using Microsoft Excel and its add-in XLSTAT 2014.

## 3. Results

The present study included 66 patients with ileal perforation peritonitis, 71.2% of whom were male. The proportion of affected individuals was higher in young adults aged 18 to 28 years and those below 18 years, with both groups contributing 28.8%. The median age of the patients was 26 years, with a range of 1 to 78 years. The median waiting time was 6 hours (1-96), and the median length of stay was 24.5 days, ranging from 3 to 102 days (Table 1).

There is a statistically significant difference (0.0348) in mean age (Table 2) between the two groups. The group of patients who did not receive enteral nutrition had a higher mean age (34.3 (19.1) years) compared to the group of patients who received early enteral nutrition (24.3 (13.7) years).

The level of delta albumin in the blood was greater in the patient cohort receiving early enteral nutrition than in the control group, as evidenced by the mean value of 34.8 (3.1) mg/l vs. 31.9 (3.6) mg/l, respectively; this difference was statistically significant (*p* value = 0.0027). Furthermore, administration of enteral nutrition led to a significantly shorter delay in peristalsis recovery compared to the unfed group with a mean duration of 2.1 (0.6) days and 3.8 (1.2) days, respectively; this difference was highly significant (*p* value < 0.0001). A significant statistical difference in the average duration of hospitalization between the two groups emerged; specifically, the control group had an average stay of 39.4 (25.3) days compared to the study group's 25.5 (14.9) days, with a *p* value of 0.0046.

The bivariate analysis showed that 22.7% of the patients enrolled in the study developed parietal infections (Table 3), of which 36.4% were in the control group (compared to 9.1% of those who received early enteral nutrition, *p* = 0.0082). The rate of fistula development in patients who did not receive early enteral nutrition was significantly higher at 28.1% (compared to 3.4% in patients who received early enteral nutrition, *p* value = 0.009). Evisceration was observed as a complication in 8% of the patients. This problem was observed in more than 24.2% of patients who did not receive early enteral nutrition compared to those who received early enteral nutrition who reported no such problem (*p* value, 0.0113). Malnutrition was significantly more common in patients who did not receive early enteral feeding, with 84.8% experiencing malnutrition compared to 21.2% of those who received early enteral feeding (*p* value less than 0.0001). Reintervention occurred in 30.3% of cases, with a significantly higher risk in patients who did not receive enteral nutrition (51.5%) compared to those who did (9.1%), with a *p* value of 0.0003.

Early enteral nutrition significantly (*p* = 0.0278) reduces the time taken for peristalsis to recover (Table 4). The adjusted odds ratio for this effect is 0.3 (95% confidence interval (CI) 0.1 to 0.9). Additionally, enteral nutrition reduces the incidence of undernutrition (*p* = 0.0039), with an odds ratio of 0.1 (95% CI 0 to 0.4).

## 4. Discussion

The geographical and financial obstacles hindering the access to artificial enteral or parenteral nutrition products used to address nutritional deficiencies causing complications (such as delayed healing, slow postoperative rehabilitation, and lengthy hospital stay) after abdominal surgery pose a significant issue in low-income nations. The use of a protein-energy solution based on locally available crop grains would serve as a palliative solution. This study evaluates the clinical effectiveness of administering locally prepared products for early enteral nutrition in patients undergoing surgery due to acute peritonitis caused by perforated ileum. The local preparation comprises soybean meal (75 g/day), maize meal (100 g/day), white rice meal (100 g/day), sugar (15 g/day), and pineapple (100 g/day).

The results of this study highlight the importance of timely enteral feeding with locally sourced nutrients in improving nutritional status and hastening peristalsis recovery.

Out of a group of 66 patients who had acute ileal perforation peritonitis, men were more prevalent than women with a ratio (M/F) of 2 : 5. Many studies [15, 16] have shown a clear male predominance in peritonitis patients. With the exception of one study from Nigeria that reported high peritonitis prevalence in women ([17] mentioned in [18]), this trend of male predominance is well-documented. Nonetheless, male patients may be at an increased risk of ileal perforation due to genetic predisposition [18]. Most patients were young, with a median age of 26 years (range: 1–78). Several African studies [18, 19] have shown a predominance of peritonitis among the young population.

The comparison of average ages between the group of patients who received early enteral nutrition and those who did not receive such nutrition revealed a statistically noticeable difference (*p* = 0.0348, Student's *t*-test). The mean age (standard deviation) was higher in the group of patients who did not receive early enteral nutrition (34.3 ± 19.1 years) compared to those who received it (24.3 ± 13.7 years). This discrepancy in age may be attributed to sampling variations. The results of the multivariate analysis indicate that age has no effect on the occurrence of predictive factors.

The median albumin level upon admission was 31 mg/dl, with fluctuations ranging between 24 and 40 mg/dl. This demonstrates that patients seek medical treatment later, when suffering from chronic undernourishment, as typhoid ileal perforation develops in the third stage [18].

The patients who received early enteral feeding showed higher albumin levels, at 34.8 (3.1) mg/dl versus 31.9 (3.6) mg/dl in the control group (*p* value, 0.0027). This elevation in albumin levels reflects the biological advantages of early feeding with locally sourced products. The pathophysiology of paralytic ileus involves local inflammation, both endogenous and exogenous opioids, and stimulation of the sympathetic system. Paralytic ileus is characterised by a combination of two (2) or five (5) of the following symptoms: nausea and vomiting, inability to tolerate solid or semiliquid food during the last 24 hours, absence of gas or stool for the past 24 hours, abdominal distension, and radiological evidence of ileus [20, 21]. The fear of symptomatic occurrences leads to oscillation in surgeons' decision to introduce enteral nutrition. Conversely, the study's findings suggest that patients who receive enteral nutrition within 48 hours experience earlier peristalsis (2.1 (0.6) days compared with 3.8 (1.2) days in patients not provided with early feeding; *p* value < 0.0001, based on Student's *t*-test). Boelens et al. [15] have also observed the benefits of enteral feeding in reducing ileus, reporting a faster initial bowel movement. As in our study, the shortened recovery time of peristalsis did not affect the occurrence of gastrointestinal symptoms such as vomiting and diarrhoea, which would be of concern in this context.

The bivariate analyses indicate that parietal infection developed in only 36.4% of patients who were enteral fed compared to 9.1% of nonenterally fed patients (*p* = 0.0082). The risk of developing enteral fistula was considerably lower in patients who received early enteral feeding at 3.4% (vs. 28.1% of those who did not receive enteral nutrition, *p* value = 0.009). The incidence of evisceration was significantly higher among patients who were not fed through enteral feeding within hours, with 24.2% affected, as compared to those who received early enteral feeding (0%), at a *p* value of 0.0113. Patients who did not receive early enteral feeding were at a significantly higher risk of undernutrition, at 84.8%, compared to those who did receive enteral feeding (21.2%). Additionally, patients who did not receive early enteral nutrition had a higher risk of requiring reintervention.

A reduction in the occurrence of parietal infection and enteral fistula was observed in the early enteral feeding group with *p* values of 0.082 and 0.00129, respectively. Additionally, Boelens et al. (2014) reported a reduced risk of enteral fistula among early enteral fed patients. Although septic shock appeared to be more common in the nonenterally fed group, there was no statistically significant association (*p* = 0.1487) compared to those receiving early enteral feeding.

A systematic review [22] indicates that five studies reveal a trend towards lower sepsis rates in the enteral feeding group when compared to the standard care group. Moreover, the resting of the digestive tract can cause intestinal atrophy, increasing the risk of bacterial translocation, diarrhoea, and decrease in intestinal immunity through reduced stimulation of the “gut-associated lymphoid tissue (GALT).” Enteral nutrition, which is less invasive and more in line with physiological processes, does not cause these harmful effects and enhances the gut and overall immunity of patients in a state of stress [23].

The hospital stay duration was significantly reduced (*p* = 0.0046) among the cohort receiving early enteral nutrition compared to those who did not receive the treatment. Furthermore, a separate research [15] provides evidence of superior outcomes for early enteral nutrition on the hospital stay duration.

Early enteral nutrition has been widely discussed in the literature [4, 22] as a critical aspect of the Enhanced Recovery After Surgery (ERAS) procedures. Precocious enteral nutrition is recommended to achieve this objective. The perioperative period requires the use of various techniques to reduce surgical stress and facilitate recovery. These techniques include preoperative preparation and premedication, maintenance of fluid balance, postoperative anaesthesia, and analgesia. Logistic regression analysis in the current study suggests that early enteral nutrition contributes to a better postoperative prognosis in perioperatively prepared patients. The benefits included a reduction in the duration of intestinal ileus and an improvement in patients' nutritional status, with odds ratios of 95% CI 0.3 (0.1, 0.9), *p* value 0.0278, and 0.1 (0, 0.4),*p* value 0.0039, respectively.

## 5. Conclusion

Surgery for acute generalised peritonitis is prone to many postoperative complications in our setting. The risk of these complications could be reduced by early enteral feeding in combination with other therapeutic measures. The use of a locally produced protein-energy ration (soya, maize, white rice, and pineapple) as early enteral feeding shows clinical benefits in improving the patients' nutritional status and postoperative recovery. These benefits suggest that manufactured locally enteral nutrition could be used as a substitute for commercially available enteral feeds, which are inaccessible in the study area. Comparative studies between commercialised enteral rations and locally prepared rations using protein-energy foods available on market in the study region are needed before promoting its use in surgical patients.

### 5.1. Strengths and Limitations of the Study

The study has two strengths:

- Firstly, it recommends early enteral feeding using a protein-energy food ration consisting of locally available products which could enhance the nutritional status and postoperative recovery of patients suffering from acute generalised peritonitis due to ileal perforation
- Secondly, the study takes place in a context of limited resources in Bukavu, in the Democratic Republic of Congo

Despite these, the study has three limits:

- The results are based on a small sample size of only 66 patients, which limits the statistical power and generalisability of the findings
- No in-depth biochemical research was conducted to assess the effects of enteral feeding on inflammatory, immune, and metabolic parameters
- Similarly, no physicochemical, organoleptic, or microbiological analyses were performed to ensure product quality and safety, assess patient preference and acceptability, or estimate the shelf life based on microbial quality

To address these issues, a forthcoming investigation ought to encompass a wider group of participants and conduct a larger quantity of clinical and biochemical assessments to determine the nutrients consumed and the physiological impacts of enteral feeding. Additionally, it should appraise the quality and suitability of the product used for enteral feeding.

## Figures and Tables

**Table 1:** Sociodemographic data and laboratory results at admission.

Variables	*n* (%)
Sex (*N* = 66)	
Female	19 (28.8)
Male	47 (71.2)
Age, years (*N* = 66)	
Under 18	19 (28.8)
18–28	19 (28.8)
29–38	12 (18.2)
39–48	7 (10.6)
49–60	5 (7.6)
Over 60	4 (6.1)
Age, median [min–max]	26 [1-78]
BMI^∗^, median [min–max] (kg/m^2^)	20.6 [16-24.2]
Waiting time to surgery, median [min–max] (hour)	6 [1-96]
Hospital stays, median [min–max] (day)	24.5 [3-102]
Number of perforations, median [min–max]	2 [1-3]
Delay in the return of peristalsis, median [min–max] (day)	2.5 [1-6]
Ionogram and blood metabolite status at admission	
Na+^∗^, median [min–max] (mg/dl)	152.5 [132-164]
K+^∗^, median [min–max] (mg/dl)	5 [3.7-8.9]
Calcium^∗^, median [min–max] (mg/dl)	2 [1.82-218]
C-reactive protein^∗^, median [min–max] (mg/l)	36 [12-124]
Albumin^∗^, median [min–max] (mg/dl)	31 [24-40]
Creatinemia^∗^, median [min–max] (mg/dl)	189.5 [106-87]
Urea^∗^, median [min–max] (mg/dl)	6.1 [2.8-16]

^∗^At the entrance.

**Table 2:** Comparison of the means between the intervention group and the control group.

Variable	Intervention group	Control group	*p*
Age (year)	24.3 (13.7)	34.3 (19.1)	0.0348
C-reactive protein (mg/l)	42.7 (25.9)	43.9 (28.4)	0.7032
BMI (kg/m^2^)	20.3 (2.4)	20.4 (2.3)	0.8675
Delta albumin^∗^ (mg/dl)	34.8 (3.1)	31.9 (3.6)	0.0027
Waiting time before intervention (hour)	15.6 (21.3)	16 (19.8)	0.9223
Number of perforations	1.7 (0.8)	1.9 (0.7)	0.3408
Delay in of peristalsis recovery (day)	2.1 (0.6)	3.8 (1.2)	<0.0001
Duration of hospitalization (day)	25.5 (14.9)	39.4 (25.3)	0.0046

^∗^Delta albumin is the average of the admission and discharge albumin levels.

**Table 3:** Association between the two intervention groups and the qualitative variables of interest.

Variables	Intervention group	Control group	Total (%)	*p*	OR [95% CI]
	*n* (%)	*n* (%)	*n* (%)	*n* (%)	
Total	33 (100)	33 (100)	66 (100)		
qSOFA					
1	19 (57.6)	22 (66.7)	41 (62.1)		
2	14 (42.4)	11 (33.3)	25 (37.9)	0.4465	0.7 [0.2-1.9]
Gender					
Female	8 (24.2)	11 (33.3)	19 (28.8)		
Male	25 (75.8)	22 (66.7)	47 (71.2)	0.4147	0.6 [0.2-1.9]
Fever					
No	20 (60.6)	18 (54.5)	38 (57.6)		
Yes	13 (39.4)	15 (45.5)	28 (42.4)	0.8033	1.3 [0.4-3.4]
Asthenia					
No	17 (51.5)	6 (18.2)	23 (34.8)		
Yes	16 (48.5)	27 (81.8)	43 (65.2)	0.0045^∗^	4.8 [1.6-14.2]
Pneumonia					
No	30 (90.9)	26 (78.8)	56 (84.8)		
Yes	3 (9.1)	7 (21.2)	10 (15.2)	0.1697	2.7 [0.6-10.7]
Abdominal distension					
No	20 (60.6)	23 (69.7)	43 (65.2)		
Yes	13 (39.4)	10 (30.3)	23 (34.8)	0.4383	0.7 [0.2-1.9]
Parietal infection					
No	30 (90.9)	21 (63.6)	51 (77.3)		
Yes	3 (9.1)	12 (36.4)	15 (22.7)	0.0082^∗^	5.7 [1.5-21.2]
Vomiting					
No	27 (81.8)	29 (87.9)	56 (84.8)		
Yes	6 (18.2)	4 (12.1)	10 (15.2)	0.7330	0.6 [0.1-2.3]
Fistula					
No	32 (97)	24 (72.7)	56 (84.8)		
Yes	1 (3)	9 (27.3)	10 (15.2)	0.0129^∗^	12 [1.9-72.7]
Sepsis					
No	31 (93.9)	26 (78.8)	57 (86.4)		
Yes	2 (6.1)	7 (21.2)	9 (13.6)	0.1487	4.2 [0.9-19.2]
Under nutrition					
No	26 (78.8)	5 (15.2)	31 (47)		
Yes	7 (21.2)	28 (84.8)	35 (53)	<0.0001^∗^	20.8 [6.1-70.6]
Evisceration					
No	33 (100)	25 (75.8)	58 (87.9)	0.0048^∗^	
Yes	0 (0)	8 (24.2)	8 (12.1)		
Oedema					
No	29 (87.9)	26 (78.8)	55 (0)	0.5105	2 [0.5-7.1]
Yes	4 (12.1)	7 (21.2)	11 (83.3)		
Hydration status					
No dehydration	19 (57.6)	10 (30.3)	29 (0)		
Mild dehydration	8 (24.2)	10 (30.3)	18 (43.9)	0.1281	
Moderate dehydration	5 (15.2)	10 (30.3)	15 (27.3)		
Severe dehydration	1 (3)	3 (9.1)	4 (22.7)		
Reintervention					
No	30 (90.9)	16 (48.5)	46 (69.7)		
Yes	3 (9.1)	17 (51.5)	20 (30.3)	0.0003	10.6 [2.9-38.8]

**Table 4:** Adjusted logistic regression model of effects linked to the use of early enteral feeding with local products.

Effects associated with early enteral nutrition	*p* value	ORa [95% CI]
Constant	0.8978	
Age	0.2029	1 [0.9-1.1]
Delta albumin	0.2039	1.2 [0.8-1.7]
Number of perforations	0.7904	0.8 [0.2-2.9]
Delay in of peristalsis recovery (day)	0.0278	0.3 [0.1-0.9]
Duration of hospitalization (day)	0.8712	1 [0.9-1.1]
Reintervention	0.4307	0.4 [0-4.3]
Asthenia	0.2281	0.3 [0-2.1]
Parietal infection	0.8042	0.7 [0-8.9]
Fistula	0.9864	1 [0-20.7]
Under nutrition	0.0039	0.1 [0-0.4]

## Data Availability

The data supporting the findings of this study are available in a saved file in .XLS format from the corresponding author, JP. B. Cikwanine, upon reasonable request. To ensure anonymity, the names of the participants will be kept confidential.
